# Optimising clinical effectiveness and quality along the atrial fibrillation anticoagulation pathway: an economic analysis

**DOI:** 10.1186/s12913-019-4841-3

**Published:** 2019-12-28

**Authors:** Eoin Moloney, Dawn Craig, Nikki Holdsworth, Joanne Smithson

**Affiliations:** 1 Health Economics Group, Institute of Health & Society, Newcastle University, Baddiley-Clark Building, Newcastle, NE2 4AX UK; 2 Academic Health Science Network North East and North Cumbria, Newcastle, UK

**Keywords:** Atrial fibrillation, Warfarin, Direct oral anticoagulants, Economic analysis, Cost-effectiveness

## Abstract

**Background:**

Atrial fibrillation (AF) represents the most common sustained cardiac arrhythmia. A service evaluation was carried out at an anticoagulation clinic in Newcastle upon-Tyne to explore the efficacy of introducing self-testing of anticoagulation status for AF patients on warfarin. The analysis presented aims to assess the potential cost savings and clinical outcomes associated with introducing self-testing at a clinic in the Northeast of England, and to determine the cost-effectiveness of a redesigned treatment pathway including genetic testing and self-testing components.

**Methods:**

Questionnaires were administered to individuals participating in the service evaluation to understand the patient costs associated with clinical monitoring (139 patients), and quality-of-life before and after the introduction of self-testing (varying numbers). Additionally, data on time in therapeutic range (TTR) were captured at multiple time points to identify any change in outcome. Finally, an economic model was developed to assess the cost-effectiveness of introducing a redesigned treatment pathway, including genetic testing and self-testing, for AF patients.

**Results:**

The average cost per patient of attending the anticoagulation clinic was £16.24 per visit (including carer costs). Costs were higher amongst patients tested at the hospital clinic than those tested at the community clinic. Improvements in quality-of-life across all psychological topics, and improved TTR, were seen following the introduction of self-testing. Results of the cost-effectiveness analysis showed that the redesigned treatment pathway was less costly and more effective than current practice.

**Conclusions:**

Allowing AF patients on warfarin to self-test, rather than attend clinic to have their anticoagulation status assessed, has the potential to reduce patient costs. Additionally, self-testing may result in improved quality-of-life and TTR. Introducing genetic testing to guide patient treatment based on sensitivity to warfarin, and applying this in combination with self-testing, may also result in improved patient outcomes and reduced costs to the health service in the long-term.

## Background

Atrial fibrillation (AF) represents the most common sustained cardiac arrhythmia, affecting more than 6 million people in Europe [1]. Vascular thrombosis and embolism are major causes of morbidity and mortality. AF prevalence and its associated service pressures are increasing in part due to a growing proportion of over 65 s in the population, but also because actual AF prevalence is likely to be much higher than predicted, as many patients with AF remain undiagnosed [2].

Two types of oral anticoagulants have been licensed for use in the UK for the treatment of AF [3]: warfarin and direct oral anticoagulants (DOACs). Warfarin has a narrow therapeutic window and its impact is vulnerable to variable metabolism; a major reason for the wide variation in optimal dose. For this reason, patients on warfarin need to be routinely monitored to ensure that their international normalised ratio (INR) is within the desired range. In contrast, DOACs have a wider therapeutic window than warfarin and are marketed with “no monitoring required” status [4]. However, they cost the UK National Health Service (NHS) significantly more money than warfarin, with expenditure on anticoagulants increasing by approximately £400 million in the 2017–18 financial year due to the increased use of DOACs [4].

Currently, AF patients attending their GP will typically be prescribed either warfarin or a DOAC. However, it has been known for two decades that around 3% of the population with north European ancestry carry two CYP2C9 variants plus a variant in the vitamin K-related VKORC1, which make them exquisitely sensitive to warfarin and more liable to overdose [5–7]. Around a third of the population have one or two of these variants, making the choice of warfarin a more finely balanced decision. The EU-PACT trial, where Newcastle was one of the recruitment centres, tested the effect of pharmacogenetic-guided warfarin dosing on anticoagulation outcomes with the aid of point-of-care genetic testing, and demonstrated clinical benefits, improved quality of patient care, increased time in therapeutic range (TTR), and fewer incidents of over-anticoagulation [8].

INR monitoring services, which are used to check the impact of warfarin on the patient, have changed little in recent years despite evidence that shows self-monitoring of anticoagulation status is both clinically and cost-effective compared with standard monitoring [9, 10]. Self-monitoring has also been shown to increase TTR [11, 12].

This study aims to assess the economic impact of both of these innovations (genetic testing and self-testing of anticoagulation status for patients on warfarin), using patient data from a service evaluation carried out at a Newcastle-upon-Tyne anticoagulation clinic and an economic model developed based on published literature.

## Methods

The analysis is split into two sections:

1. An evaluation of the patient cost savings and consequences of implementing self-testing of anticoagulation status for individuals currently undergoing warfarin treatment at a Newcastle-upon-Tyne anticoagulation clinic (based on responses to patient-completed cost and quality-of-life questionnaires and data on TTR pre- and post-intervention).
2. An evaluation of the cost-effectiveness of implementing a redesigned treatment pathway (including genetic testing of warfarin sensitivity, and self-testing) compared to current practice, based on a decision-analytic model.

### Cost-consequence analysis

A cost-consequence analysis was conducted to estimate the change in patient costs and outcomes (TTR and quality-of-life) after undergoing self-testing. All patients on warfarin receiving monitoring at the anticoagulation clinic were asked if they would like to undertake self-testing as part of the service evaluation. Where patients agreed, they received initial training and a competency check from a clinic nurse 1 week later to ensure that they could be signed off as competent to self-test. Baseline data on patient costs and quality-of-life were collected at initial training, before the competency check was carried out and prior to the patient beginning self-testing.

#### Patient costs

A questionnaire to estimate patient costs of attending the anticoagulation clinic (time and travel questionnaire) was developed and administered to patients at the clinic (Additional file 1). Responses to the questionnaire could be used to estimate the average patient cost of attending clinic and the cost savings that could be made by patients as a result of reducing their attendance at clinic.

For costs of patient (and carer) time, the time that they have indicated that they spend travelling to and from the anticoagulation clinic, as well as the time spent at the clinic, was combined with information on the type of activity, i.e. employment/leisure etc., that was displaced by accessing care. Hourly costs for these activities were derived from routine sources for working and non-working time [13, 14]. The cost of time was valued at the national median wage rate per hour if the patient (and/or carer) missed paid work [13], while the cost of leisure time was valued at the Department of Transport cost per hour of leisure time [14]. Out-of-pocket monetary costs (if applicable) for accessing care were taken directly from responses to the questionnaire where the patient (and/or carer) travelled by public transport and, where the patient (and/or carer) travelled by car, monetary costs were estimated by combining information on the distance travelled with the cost of travel per mile, derived from routine sources [14]. Total cost of accessing care per patient could then be estimated. Costs were estimated for a 2018 price year (£).

#### Quality-of-life

A quality-of-life questionnaire (Sawicki, 1999 [15]) was administered to patients at baseline and 6 months after beginning self-testing to understand changes in patient satisfaction pre- and post-intervention (Additional file 2). The Anticoagulation Treatment (Warfarin) Questionnaire is a 32-item measure that has been used to measure differences in quality-of-life between patients who receive warfarin and DOACs and between patients who undergo clinical examination and self-management of warfarin treatment [15]. Each of the 32 individual questions is scored from one (total disagreement) to six (total agreement) and groups of individual statements are combined into five broad psychological topics: (1) Medical treatment satisfaction, (2) Self-efficacy, (3) General psychological distress, (4) Daily hassles, and (5) Strained social network. A mean score of between one and six can then be estimated for each of the individual topics. Improvements in quality-of-life are indicated by higher scores for the ‘Medical treatment satisfaction’ and ‘Self-efficacy’ topics, and by lower scores for the ‘General psychological distress’, ‘Daily hassles’ and ‘Strained social network’ topics. Mean scores for each topic were estimated at baseline and at 6 months. The mean difference in score for each psychological topic was then estimated, and a paired samples t-test was carried out to look at the statistical significance of any differences in scores.

#### TTR

Finally, data on TTR (percentage of time in past 6 months in which INR was in target range) were collected from the DAWN AC System on all patients undergoing self-testing. TTR was calculated using the Rosendaal method, which looks at two INRs at a time and calculates the proportion of the line that is currently in range (each individual has their own personal target INR range) [16]. These data were collected three, and six, months pre-intervention and 6 months post-intervention, and were analysed to understand the changes in TTR outcomes (percentage of TTR) for patients undergoing self-testing.

Cost and clinical data were analysed and presented at an average level. Analyses were carried out using Stata 15 statistical software. Data from as many patients as possible were collected on the different outcomes at relevant time points. This led to some variability in the number of patients included in each outcome analysis.

### Cost-effectiveness analysis

This section describes the long-term economic model developed to estimate the costs and effects of introducing a redesigned treatment pathway (including genetic testing and self-testing) for AF patients, compared with current clinical practice. The model was designed and populated based on information from published literature. A description of the included model parameters can be seen in Additional file 3. The CHEERS reporting guidelines were followed in presenting details of the economic model [17].

#### Model description

An economic model, developed as a Markov cohort model, was built in TreeAge Pro® (TreeAge Software, Inc., Williamstown, MA, USA) [18] to estimate the cost-effectiveness of introducing the full redesigned pathway for management of AF patients, compared with current clinical practice. The population considered was patients with atrial fibrillation aged 65 and over. The model structure is shown in Additional file 4: Figure S1.

Patients begin the model at a point where they can receive either current patient management or redesigned management. Where patients receive current practice, they are initially prescribed either warfarin or a DOAC (DOAC assumed to be dabigatran based on clinical evidence available for this medication, although prescribing practices vary across localities). The clinical pathways following each choice of treatment are identical. All patients are initially assumed to be well on treatment, and from this health state patients can go on to experience an ischaemic stroke, myocardial infarction (MI), major bleeding event, systemic embolism, or they may die. Alternatively, patients may not experience any adverse event and therefore, remain in the same health state. Depending on the events experienced during this clinical pathway, patients move on to subsequent health states ‘post stroke with no deficit’, ‘post stroke with mild deficit’, ‘post stroke with major deficit’, depending on the severity of the original non-fatal ischaemic stroke, ‘post MI’ following a non-fatal MI, ‘post major bleed/intracranial haemorrhage (ICH)’ following a non-fatal ICH, or ‘Dead’ following a fatal clinical event or from general background mortality. Where patients do not experience any adverse clinical event, or experience a systemic embolism or extracranial haemorrhage and survive, they return to the ‘Well’ health state. In all post event health states patients can either survive or die, with the mortality risk increased following a stroke or major bleed.

Where patients follow the redesigned pathway, they begin by receiving a genetic test and are grouped as having major sensitivity, moderate sensitivity or normal sensitivity (to warfarin) based on the results of this test. Patients then follow the same care pathways as described previously, i.e. are prescribed either warfarin or a DOAC and can go on to experience various adverse clinical events, die or remain well.

#### Model assumptions

1. Patients on warfarin undergoing clinical monitoring with current practice are assumed to attend 11 clinic visits over the first 3 months (dose initiation stage), and subsequently attend the clinic once per month for monitoring.
2. In the redesigned pathway, it is assumed that the percentage of patients receiving warfarin varies depending on genetic test results. Therefore, patients with normal sensitivity have a higher probability of receiving warfarin than those with moderate and major sensitivity.
3. It is assumed that all warfarin patients in the redesigned pathway are self-testing, regardless of sensitivity status. It is assumed that these patients are clinically monitored in the first 3 months following a genetic test and that from this point onwards they self-test only. This clinical monitoring in the first 3 months following a genetic test is assumed to be 75% the cost of clinical monitoring with current practice, as genetic testing would result in the dose being stabilised quicker, as per Janzic et al. [19]. However, in order to account for the fact that patients with genotypes of high or low warfarin sensitivity would be managed by intensified anticoagulation care (monitoring at least twice per month, rather than the monthly monitoring assumed with current practice), in the model it is assumed that in the first 3 months patients with moderate or major sensitivity receive bi-monthly monitoring for the first 3 months, before beginning self-testing.
4. It is assumed that the risk reduction for the different clinical complications associated with self-testing is the same, regardless of sensitivity status of the patient.
5. It is assumed that in the redesigned pathway, warfarin patients continue to self-test even after experiencing an adverse event, other than following a major bleed or intracranial haemorrhage, in which case patients switch to taking aspirin (current practice and redesigned pathway).
6. Relative risks of experiencing a bleed or thromboembolic event have been applied to the clinical probabilities for patients on warfarin in the redesigned pathway to account for the fact that patients are self-testing. These relative risks are applied to the stroke, MI and systemic embolism probabilities.
7. In the redesigned pathway, probabilities of experiencing a stroke and major bleed are based on time in range and out of range and these values vary at the three levels of sensitivity to warfarin (normal/moderate/major). However, in the redesigned pathway, the probabilities of experiencing an MI or systemic embolism have not been estimated in the same way, as these probabilities are set in the usual care arm, i.e. not based on time in range and out of range.
8. In the redesigned pathway, patients on DOACs follow the same pathway and have the same likelihood of clinical events as patients on DOACs in the current practice pathway. The only difference between these patients is that in the redesigned pathway, all patients initially receive a genetic test. As with current practice, patients in the redesigned pathway on a DOAC who survive stroke when on dabigatran 110 mg switch to dabigatran 150 mg twice daily. Patients who experience a major bleed or intracranial haemorrhage on DOACs switch to aspirin (as is also the case when on warfarin).

#### Assessment of costs and consequences

The model was run to obtain the expected values for each strategy over the patient lifetime. The analysis was designed to generate the incremental cost per quality-adjusted life-year (QALY) gained. All costs were for a 2018 price year (£), and were inflated to this price year where appropriate. Where available, and where appropriate, data were entered into the model as distributions in order to fully incorporate the uncertainty around parameter values so that a probabilistic sensitivity analysis (PSA) could be undertaken. The PSA was run with 10,000 simulations and probabilistic output was produced. Deterministic sensitivity analyses were also conducted to explore individual, and multiple, parameter variation and the impact that this would have on the results. All costs and health effects were considered from a UK NHS and personal social services (PSS) perspective and were discounted at an annual rate of 3.5% [20].

## Results

### Cost-consequence analysis

#### Patient costs

The average cost of attending the warfarin clinic was estimated based on patient responses to the ‘Cost of attending anticoagulation clinic’ questionnaire (Additional file 1). Patient data for 139 patients were included in the analysis. Costs of attending the anticoagulation clinic are shown in Table 1. Based on all responses received, the average cost per patient of attending the anticoagulation clinic was £13.86 per visit, with this cost increasing to £16.24 per patient when the cost of the carer’s time was also included. Only 22 patients in total reported that they required help in travelling to the clinic. Respondents were also asked to indicate how often they have their blood tested, and using this information along with the average cost per visit, the average monthly cost per patient of attending clinic could be estimated. These costs were £16.43 per month excluding carer costs, and £18.75 including carer costs. Over 1 year, this would translate to savings of £197.16 per patient excluding carer costs, and £225.00 including carer costs, were self-testing to be introduced for AF patients on warfarin.

**Table 1:** Patient costs of attending anticoagulation clinic

Variable	Mean (£) (Standard deviation (£))
Cost per patient per visit	13.86 (11.47)
Cost per patient per visit (including carer costs)	16.24 (14.29)
Monthly cost per patient	16.43 (29.21)
Monthly cost per patient (including carer costs)	18.75 (29.75)

A subgroup analysis was also conducted to compare costs incurred by patients undergoing testing at the hospital clinic with patients undergoing testing at the community clinic. Individual visit, and monthly, costs were higher amongst patients who underwent testing at the hospital clinic (£15.11 and £20.20, respectively (without carer costs)) compared to those who attended the community clinic (£12.21 and £11.14, respectively).

#### Quality-of-life

The quality-of-life of all patients was estimated at baseline and at 6 months following the introduction of the self-testing intervention. Average quality-of-life scores for the five individual psychological topics, at baseline and 6 months, as well as the minimum and maximum average scores identified, are presented in Table 2 below. A varying number of patients completed all questions for each of the psychological topics, and this variation is also shown. The number of patients who completed the questionnaire at baseline was greater than the number who completed the questionnaire at 6 months, primarily because many patients had not yet reached 6 months self-testing upon completion of the evaluation. Additional numbers may then have been missing at each time point for each of the psychological topics due to failure to complete specific questions on each of the topics. The statistical significance of any differences in mean scores was tested using the paired samples t-test, with the null hypothesis being that the difference between mean scores is zero. The null hypothesis could be rejected where the associated *p*-value was less than 0.05, indicating a statistically significant difference in scores. Results of the t-test were based on the total number of patients who answered all questions related to each psychological topic at the two time points.

**Table 2:** Quality-of-life scores

Psychological Topics	Number of patients	Baseline mean (SD)	Min	Max	Number of patients	6-month mean (SD)	Min	Max	Difference compared to baseline	Paired samples t-test *p*-value (n patients)
Medical treatment satisfaction	119	4.97 (1.08)	1	6	88	5.60 (0.55)	3.6	6	+ 0.63	0.00 (72)
Self-efficacy	125	4.40 (1.40)	1.25	6	88	4.61 (1.32)	2.25	6	+ 0.21	0.11 (76)
General psychological distress	124	2.32 (1.07)	1	5.43	87	2.09 (0.91)	1	4.71	- 0.23	0.03 (74)
Daily hassles	117	2.34 (0.83)	1	5	85	2.23 (0.78)	1	4.13	- 0.11	0.15 (70)
Strained social network	126	1.72 (0.88)	1	4.75	88	1.52 (0.63)	1	3.75	- 0.2	0.01 (77)

Minimum score = one, maximum score = six. Mean, min and max scores are calculated based on responses from patients who completed all relevant questions for a given psychological topic

Results indicate that at 6 months, following the introduction of self-testing, the mean scores for medical treatment satisfaction and self-efficacy are higher than at baseline (+ 0.63 and +  0.21, respectively) while the mean scores for general psychological distress, daily hassles and strained social network are all lower than at baseline (− 0.23, − 0.11 and – 0.20, respectively). For the medical treatment satisfaction, general psychological distress, and strained social network psychological topics, we can reject the null hypothesis that the difference between mean scores is zero, i.e. *p*-value < 0.05. Therefore, there is a statistically significant difference between the scores.

In a separate analysis, differences in quality-of-life between patients monitored at the hospital clinic and patients monitored at the community clinic were also assessed at baseline. Improved quality-of-life scores for the medical treatment satisfaction, strained social network, self-efficacy and daily hassles topics were seen for patients monitored at the community clinic, while for the general psychological distress topic, quality-of-life was higher for patients monitored at the hospital clinic.

#### TTR

TTR percentage prior to, and following, the introduction of self-testing for warfarin patients, and change in TTR percentage, are presented in Tables 3 and 4 below. Data on TTR were collected 3 and 6 months before the implementation of self-testing, and 6 months following its introduction. Data for 135 patients were collected 6 months prior to the introduction of self-testing and the average TTR score was 71%. Data for 137 patients were collected 3 months prior and the average TTR score was 73%. For 109 of these patients, data 6 months following the introduction of self-testing were collected and the average TTR score was 75%. For 105 patients, data were collected both 6 months prior to and 6 months following the introduction of self-testing (32 of the patients included in the dataset had not yet reached the point at which they would require the 6 month test). The average increase in percentage of TTR for those patients for whom data were collected 6 months before and after the introduction of self-testing was 3%. For 108 patients, data were collected 3 months prior to and 6 months following the introduction of self-testing. The average increase in percentage of TTR for those patients for whom data were collected 3 months before and 6 months after the introduction of self-testing was 0.5%.

**Table 3:** TTR % prior to, and following, the introduction of self-testing

Variable	Mean (%) (SD (%))
TTR 6 months prior	71 (23)
TTR 3 months prior	73 (22)
TTR 6 months following	75 (17)

**Table 4:** Difference in TTR % prior to, and following, the introduction of self-testing

Variable	Mean change (%) (SD (%))
Difference between TTR 6 months prior and 6 months following introduction of self-testing	+ 3 (23)
Difference between TTR 3 months prior and 6 months following introduction of self-testing	+ 0.5 (23)

### Cost-effectiveness analysis

This section presents the results of the cost-effectiveness analysis. Results of the base-case analysis are presented first, followed by results of the sensitivity analyses.

#### Base-case analysis

The base-case results indicate that the redesigned treatment pathway is cost-effective, and that current practice is absolutely dominated, i.e. less effective (−0.01) and more costly (+£1397). Results from the PSA show that the redesigned treatment pathway has a higher probability of being cost-effective across a range of willingness-to-pay (WTP) thresholds (Additional file 5: Figure S2). The cost-effectiveness plane (Additional file 6: Figure S3) also shows that although the majority of points are clustered around zero cost and zero effect, there is a marginally higher number of points in the south-east quadrant (indicating that the intervention is more effective and less costly).

#### Sensitivity analysis

A number of sensitivity analyses were also carried out to explore the impact that key parameter variation (with all other parameters fixed at base-case values) had on the model results (Table 5). Firstly, the impact of varying the percentage of patients who are prescribed warfarin in current practice was explored. When this probability was decreased to 0.1 (i.e. 90% of patients prescribed DOAC), current practice was found to be even more expensive relative to the redesigned pathway. However, in this scenario current practice was now more effective (+ 0.08) than the new treatment pathway (although not cost-effective based on a maximum WTP threshold of £40,000). When this probability was increased to 0.9, the redesigned pathway was now more expensive than current practice but even more effective (+ 0.19) than in the base-case analysis. The resulting incremental cost-effectiveness ratio (ICER) in this scenario analysis was £8195, meaning that the redesigned treatment pathway would be considered cost-effective.

**Table 5:** Sensitivity analyses

Analysis	Cost difference vs. current practice (£)	QALY difference vs. current practice	ICER for ‘redesigned treatment pathway’ (cost per QALY gained) (£)
Base-case result	−1397	0.01	Dominant
Sensitivity analysis			
Probability of taking warfarin with current practice (base-case 0.4)			
0.1	− 3285	−0.08	42,040^a^
0.9	1522	0.19	8195
Reduction in probability of having moderate sensitivity in redesigned pathway (base-case 0.27) (increasing probability of having normal sensitivity)			
0.20	− 1693	0.03	Dominant
0	− 2322	0.06	Dominant
Increase in probability of having major sensitivity in redesigned pathway (base-case 0.07)			
0.15	− 1081	0.02	Dominant
0.6	1087	0.04	30,139
Decrease in probability of taking warfarin if normal sensitivity in redesigned pathway (base-case 0.9)			
0.5	50	0.05	1089
0.1	1585	0.07	23,012

^a^ICER for current practice

Separate exploratory analyses were conducted to explore the impact of increasing the percentage of patients with normal sensitivity, and major sensitivity, to warfarin following genetic testing in the redesigned treatment pathway. None of these variations changed the base-case decision. Finally, on the basis of clinical advice (clinical experts involved in the study), the percentage of patients with normal sensitivity to warfarin who are prescribed warfarin following a genetic test may not be as high as 90%, as assumed in the base-case analysis. Therefore, this probability was decreased to 0.5 and 0.1 in two separate sensitivity analyses. Following these variations, the redesigned pathway was more expensive than current practice (with cost of the new pathway increasing as this probability is lowered), while effectiveness also increased. In both scenarios, the redesigned treatment pathway remained cost-effective.

## Discussion

An economic analysis was carried out to explore the costs and health outcomes associated with the introduction of two innovations (genetic testing of warfarin sensitivity and self-testing of anticoagulation status) for the management of patients with atrial fibrillation. Two separate analyses were conducted; a cost-consequence analysis focussing on self-testing, using patient data collected at Newcastle-upon-Tyne anticoagulation clinic, and a cost-effectiveness analysis of a redesigned treatment pathway, informed by the literature.

Results from the cost-consequence analysis highlight the cost savings that could be made by patients on warfarin by introducing self-testing as a replacement for clinical monitoring of anticoagulation status. An average saving of £18.75 per patient per month (including carer costs) could be made by patients by eliminating the requirement for patients to travel to the anticoagulation clinic to be monitored. Costs are incurred through patients having to take time off work and other activities, travel to clinic and pay out of pocket for parking and public transport (if applicable), and the introduction of self-testing can remove these costs. The results also highlight the fact that costs incurred are higher for patients who attend the hospital clinic compared to those who attend the community clinic due to the fact that patients attending the hospital clinic will typically have to travel longer distances, will often find it more difficult to find parking and will regularly have to wait longer than patients who attend the community clinic (many of whom will be seen at a specific appointment time). In our sample, 58% of patients underwent monitoring at the hospital clinic. Although in practice, data indicate that most patients are in fact monitored at the community clinic, these results highlight the cost savings that could be made by reducing the percentage of patients monitored at the hospital clinic even further. Clinical results from the cost-consequence analysis also show an average improvement in quality-of-life scores (across all psychological topics), and an average increase in time in therapeutic range for patients following the introduction of self-testing.

A cost-effectiveness analysis, informed by the literature, was also conducted to explore the long-term costs and outcomes associated with the introduction of a full redesigned pathway for patients receiving treatment for atrial fibrillation. Base-case probabilistic results show the high likelihood of the new pathway being cost-effective across all willingness-to-pay thresholds. Additionally, multiple sensitivity analyses were carried out, none of which had an impact on the base-case decision, with the intervention strategy remaining dominant (less costly and more effective) in most. However, sensitivity analyses conducted do indicate that the percentage of patients who are categorised as having normal/moderate/major sensitivity following the genetic test, and the percentage of patients prescribed warfarin/DOAC at each level of sensitivity, are strong drivers of costs and effects.

The analysis presented here is unique in that it is the first time that real-world data from patients undergoing self-testing who were previously clinically monitored at an anticoagulation clinic in the Northeast of England, have been used in an economic analysis. Results of our cost-effectiveness analysis are in line with previous economic evaluations which have shown the potential cost-effectiveness of point-of-care tests for the self-monitoring of anticoagulation status [10]. However, our analysis is novel in that it combines both genetic testing and self-testing components in one model, which is a first for the UK setting.

There are limitations to this analysis, primarily around the uncertainty surrounding many of the parameter estimates included in the cost-effectiveness analysis. Consequently, a number of assumptions were made in developing and populating the economic model. Most notably, there was a lack of information available on how the genetic test results would impact the percentage of patients receiving warfarin as opposed to a DOAC. Additionally, in the model it was assumed that self-testing would have the impact of reducing the probability of experiencing a complication but it was unknown how the sensitivity status of the patient would impact this probability. Therefore, it was assumed that the risk reduction associated with self-testing was the same regardless of genetic test results. Where possible, these assumptions were explored in sensitivity analysis. Variation of the probability of receiving warfarin when identified as having normal sensitivity was conducted, but this variation did not result in a change in the cost-effectiveness decision. Future economic analyses in this area should attempt to use data which may remove some of the uncertainty present in the existing model. Finally, results from the cost-consequence analysis highlight the economic and clinical benefits that could result from a switch to self-testing and a cost-effectiveness model now exist for re-analysis once additional data become available.

## Conclusions

The introduction of self-testing for atrial fibrillation patients on warfarin has the potential to reduce costs for patients and improve patient outcomes, based on data collected from a service evaluation at an anticoagulation clinic in the Northeast of England. Additionally, a redesigned treatment pathway including genetic testing and self-testing components could potentially reduce costs to the health care service and improve long-term clinical outcomes for patients with atrial fibrillation.

## Supplementary information

**Additional file 1.** Understanding the cost of attending anticoagulation clinics.

**Additional file 2.** Anticoagulation Treatment (Warfarin) Questionnaire.

**Additional file 3.** Estimation of model parameters.

**Additional file 4:** **Figure S1.** Economic model to estimate the cost-effectiveness of a redesigned treatment pathway for patients with Atrial Fibrillation.

**Additional file 5:** **Figure S2.** Cost-effectiveness acceptability curve of redesigned treatment pathway vs. current practice: base-case analysis.

**Additional file 6:** **Figure S3.** Cost-effectiveness plane of redesigned treatment pathway vs. current practice: base-case analysis.

## Data Availability

The data that support the findings of this study are available from the Academic Health Science Network for the North East and North Cumbria, but restrictions apply to the availability of these data, which were used under license for the current study, and so are not publicly available. Data are however available from the authors upon reasonable request and with the permission of the Academic Health Science Network for the North East and North Cumbria.

## Abbreviations

AF: Atrial fibrillation
DOAC: Direct oral anticoagulant
ICER: Incremental cost-effectiveness ratio
ICH: Intracranial haemorrhage
INR: International normalised ratio
MI: Myocardial infarction
NHS: National Health Service
PSA: Probabilistic sensitivity analysis
PSS: Personal social services
QALY: Quality-adjusted life-year
SD: Standard deviation
TTR: Time in therapeutic range
WTP: Willingness-to-pay
