# Supplementary material for: Optimising clinical effectiveness and quality along the atrial fibrillation anticoagulation pathway: an economic analysis
Source: BMC Health Serv Res. 2019 Dec 28;19:1007. doi: 10.1186/s12913-019-4841-3 (PMC6935474; doi:10.1186/s12913-019-4841-3)
Supplement: Supplementary file 1 — Additional file 1. Understanding the cost of attending anticoagulation clinics. [file 12913_2019_4841_MOESM1_ESM.docx]

Supplementary Information 1

**Understanding the cost of attending anticoagulation clinics**

In order to understand more about the benefits of patients monitoring their warfarin at home, we would be grateful if you could complete this questionnaire.

**The information that you provide will be made anonymous and carefully protected.**

1. **What is your current employment status?**

- Full-time employment
- Part-time employment
- Full-time education (Please continue with question 8)
- Retired (Please continue with question 8) Hourly working costs 6.04
- Looking after home and/or family
- Carer for another person
- Unemployed, actively seeking work Hourly working costs 6.04
- Unemployed, not actively seeking work Hourly working costs 6.04
- Permanently unable to work owing to long-term sickness or disability
  (Please provide details in the space provided below)
- Other (please provide details in the space provided below)

_________________________________________________________________________

1. **Please answer the following questions about your main job.**

- Your main job is the job in which you currently work the most hours.
- If you are not currently in a job, your main job is your most recent job in which you worked the most hours.

**In your main job, are you (or were you):**

- An employee?
- Self-employed or freelance without employees?
- Self-employed with employees?

1. **In your main job, what is (or what was) your full and specific job title?**(For example, PRIMARY SCHOOL TEACHER, CAR MECHANIC, DISTRICT NURSE, STRUCTURAL ENGINEER… Please do not state your grade or pay band.)

**__________________________________________________________________________**

1. **Briefly describe what you do (or what you did) in your main job.**

**__________________________________________________________________________**

1. **In your main job, do you (or did you) supervise any employees?**(Supervision involves overseeing the work of other employees on a day-to-day basis.)

- Yes
- No

1. **In your main job, how many hours per week (including paid and unpaid overtime) do you (or did you) usually work?**

- 10 or less
- 11 – 15
- 16 – 20
- 20 – 24
- 25 – 29
- 30 – 34
- 35 – 39
- 40 – 44
- 45 – 49
- 50 or more

1. **Into which of the following bands does your annual household income from all sources, before tax, fit? (tick one box):**

- Please remember this information will be made anonymous and carefully protected.

| - Less than £5,200 - £5,200 - £10,399 - £10,400 - £15,599 - £15,600 - £20,799 - £20,800 -£25,999 - £26,000 - £36,399 | - £36,400 - £51,999 - £52,000 - £77,999 - £78,000 or above - I don’t know - I would rather not say |
| --- | --- |

1. **How often do you usually have your blood tested?**

| - More than once a week - Once a week - Every two weeks - Once a month | - Every two months - Every three months - Every six months - I don’t know |
| --- | --- |

1. **Where do you usually get your blood tested? Please circle**

Hospital Clinic Community Clinic Home visit by Nurse

Other (please describe) ________________________________________

1. **If you travel to a clinic, is this by: Please circle**

Car Bus Taxi Ambulance Walk Other: _____________

1. **How far do you currently travel to clinic to have your blood tested?**

Approximately ___________ miles

1. **How long does it take you to travel to the clinic?**

Approximately _____Hours _____Minutes

1. **How much does it cost you to travel to the clinic and back if you use a bus or taxi?**
   £ ________
2. **If you travel by car, how much does parking cost?** £ ________
3. **Do you need help to get to clinic?** 🞎 Yes 🞎 No
4. **If you need help to get to clinic, who attends with you e.g. Carer/relative or other?**

Please describe **_______________________________________________________**

1. **What do you think will be the greatest benefit of monitoring your warfarin at home?**

**Thank you for supporting our project evaluation.**
