# Supplementary material for: Optimising clinical effectiveness and quality along the atrial fibrillation anticoagulation pathway: an economic analysis
Source: BMC Health Serv Res. 2019 Dec 28;19:1007. doi: 10.1186/s12913-019-4841-3 (PMC6935474; doi:10.1186/s12913-019-4841-3)
Supplement: Supplementary file 2 — Additional file 2. Anticoagulation Treatment (Warfarin) Questionnaire. [file 12913_2019_4841_MOESM2_ESM.docx]

Supplementary Information 2

**Anticoagulation Treatment (Warfarin) Questionnaire**

There are a lot of personal fears connected with the anticoagulation treatment. We would like to know your thoughts and feelings during the treatment so we can give you adequate support in dealing with anticoagulation.

The following sentences originate from patients on anticoagulation treatment. Please mark spontaneously the statements which were personally most relevant to you during the last few weeks.

**Which statement applied to you during the last weeks...?**

|  |  | doesn't apply | hardly  applies | doesn't  quite apply | applies  a little | applies a great deal | applies  fully |
| --- | --- | --- | --- | --- | --- | --- | --- |
| 1 | My treatment causes me to feel worried or stressed. |  |  |  |  |  |  |
| 2 | The effort in controlling my blood coagulation values bothers me when away from home. |  |  |  |  |  |  |
| 3 | Despite my treatment I am able to organize my free time as I wish. |  |  |  |  |  |  |
| 4 | I am dissatisfied with the amount of time I have to invest in checking my blood values. |  |  |  |  |  |  |
| 5 | I feel I've learned to cope with my treatment. |  |  |  |  |  |  |
| 6 | Despite the risk of injury, I can carry out my housework without feeling restricted. |  |  |  |  |  |  |
| 7 | I avoid doing certain activities (i.e. riding a bike) because of the risk of accidents. |  |  |  |  |  |  |
| 8 | My treatment worries my relatives. |  |  |  |  |  |  |
| 9 | I can cope well with the problems related to my treatment which can occur. |  |  |  |  |  |  |
| 10 | I worry about my future health. |  |  |  |  |  |  |
| 11 | Due to the high risk of injury I'm scared of doing spontaneous physical exercise. |  |  |  |  |  |  |
| 12 | I am dissatisfied with the length of time needed before getting the results of my blood coagulation values. |  |  |  |  |  |  |
| 13 | I'm worried that my treatment could shorten my life. |  |  |  |  |  |  |
| 14 | I dislike having to plan in detail my activities in advance. |  |  |  |  |  |  |

|  |  | doesn't apply | hardly  applies | doesn't  quite apply | applies  a little | applies a great deal | applies  fully |
| --- | --- | --- | --- | --- | --- | --- | --- |
| 15 | The uncertainty that I experience while waiting for my blood coagulation results, bothers me. |  |  |  |  |  |  |
| 16 | I have less contact with friends since undergoing my treatment. |  |  |  |  |  |  |
| 17 | I avoid going on holiday because I'm unable to estimate the negative effects the different food could have on my treatment results. |  |  |  |  |  |  |
| 18 | I'm well informed as to what I must do in order to achieve a blood coagulation value within target limits. |  |  |  |  |  |  |
| 19 | I feel dependent on my anticoagulant medication. |  |  |  |  |  |  |
| 20 | I avoid travelling as I'm afraid of not receiving adequate treatment, in case of my blood coagulation value being too low or too high. |  |  |  |  |  |  |
| 21 | I'm fed up with the amount of time spend on visiting the doctor. |  |  |  |  |  |  |
| 22 | I would take a more active interest in sport if I didn't have to take anticoagulants. |  |  |  |  |  |  |
| 23 | I have problems at work because I'm often absent (due to my treatment). |  |  |  |  |  |  |
| 24 | Sometimes I'm not sure if I can cope with my treatment. |  |  |  |  |  |  |
| 25 | I tend to worry about things. |  |  |  |  |  |  |
| 26 | Despite regular visits to the doctor, I don't feel restricted. |  |  |  |  |  |  |
| 27 | It upsets me that most people don't understand the problems connected with my treatment. |  |  |  |  |  |  |
| 28 | When going to the dentist or other doctors, I'm worried about them not knowing enough about anticoagulation. |  |  |  |  |  |  |
| 29 | My treatment has affected my sex life. |  |  |  |  |  |  |
| 30 | It bothers me being treated as an invalid. |  |  |  |  |  |  |
| 31 | I am worried about the side effects anticoagulants could have on me. |  |  |  |  |  |  |
| 32 | I worry about how others react to my treatment. |  |  |  |  |  |  |

**Thank you for your support**
