# Supplementary material for: Optimising clinical effectiveness and quality along the atrial fibrillation anticoagulation pathway: an economic analysis
Source: BMC Health Serv Res. 2019 Dec 28;19:1007. doi: 10.1186/s12913-019-4841-3 (PMC6935474; doi:10.1186/s12913-019-4841-3)
Supplement: Supplementary file 3 — Additional file 3. Estimation of model parameters. [file 12913_2019_4841_MOESM3_ESM.docx]

Supplementary Information 3

***Estimation of model parameters***

The parameters and sources used in the long-term model are summarised in Table 1-Table 4. A range of data were required to populate the model. Parameter estimates reported in the published literature were identified through a review of model-based economic evaluations and were also sourced from peer-reviewed clinical papers, and through expert clinical advice (clinical experts involved in the study). The values used in the sensitivity analysis aim to test all model assumptions and uncertainty around the base-case model parameter estimates.

**Resource use and costs**

Costs of medication were sourced from routine UK sources [1]. The 3-monthly cost of warfarin treatment was based on a 10mg dose taken on day 1 and 5mg per day taken for the rest of time. This dose is consistent with that recommended in the EU-PACT trial [2], which states that a loading dose is recommended for patients on day 1, 5mg on days two and three and doses on days four and five and thereafter determined according to local clinical practice. The 3-monthly cost of dabigatran was based on a 110/150mg dose taken twice daily. The 3-monthly cost of aspirin was based on a 75mg dose taken once daily. The cost of carrying out the genetic test was derived from a previous cost-effectiveness analysis [3], based on the average cost of a number of different assays, and included the cost of the test, consumables, labour and reagent costs. The 3-monthly cost of clinically monitoring stable and unstable warfarin patients was derived from a previous UK-based systematic review and cost-effectiveness analysis [4]. The warfarin monitoring cost for stable patients was based on 12 monitoring visits per year, with an assumed doubling of costs for unstable patients. However, based on ‘Guidelines for the Management of Adult Patients requiring Anticoagulation Treatment with Warfarin’ produced by the Newcastle upon Tyne Hospitals NHS Foundation Trust [5], in the first three months of dose initiation, warfarin patients were assumed to undergo 11 monitoring visits in total. The cost of monitoring DOAC patients was based on the cost of a creatinine blood test (£3.02) and the cost of a GP consultation (£37) (assumed to take place annually for patients on DOACs) [6, 7]. Separate costs were included to account for the cost of monitoring warfarin patients in the three months following a genetic test (and prior to initiating self-testing), with these costs adjusted depending on whether the patient had normal, moderate or major sensitivity (to warfarin), as informed by previous literature [8]. The 3-monthly cost of self-testing for warfarin patients in year 1 and in subsequent years was derived from the UK-based systematic review and cost-effectiveness analysis [4] and included the cost of the device and training to carrying out self-testing. Finally, the acute costs of adverse events and 3-monthly chronic event costs were derived from previous literature and routine UK sources [4, 9, 10]. Where required, data were inflated to a 2018 price year. All costs included in the model are shown in Table 1 below.

***Table 1*** ***Unit costs used in the model***

| **Variable** | **Value in model (£)** | **Distribution** | **Source** |
| --- | --- | --- | --- |
| Warfarin 5mg (3-monthly) | 1.92 (Total cost of warfarin sodium 5mg x 28 = £0.59; AAH Pharmaceuticals) | Gamma, Mean = 1.92, Standard deviation = 1.92 | British National Formulary, 2018 [1] |
| Dabigatran 110mg (3-monthly) | 153 (Total cost of dabigatran etexilate 110/150mg x 60 = £51.00; Pradaxa) | Gamma, Mean = 153, Standard deviation = 153 | British National Formulary, 2018 [1] |
| Dabigatran 150mg (3-monthly) | 153 (Total cost of dabigatran etexilate 110/150mg x 60 = £51.00; Pradaxa) | Gamma, Mean = 153, Standard deviation = 153 | British National Formulary, 2018 [1] |
| Aspirin 75mg (3-monthly) | 3.63 (Total cost of aspirin 75mg x 28 = £1.13; AAH Pharmaceuticals) | Gamma, Mean = 3.63, Standard deviation = 3.63 | British National Formulary, 2018 [1] |
| Genetic testing | 124.97 | Gamma, Mean = 124.97, Standard deviation = 124.97 | You et al. (2012) [3] |
| Monitoring stable warfarin patients (3-monthly) | 44.12 | Gamma, Mean = 44.12, Standard deviation = 44.12 | Sharma et al. (2015) [4] |
| Monitoring unstable warfarin patients (3-monthly) | 88.24 | N/A | Sharma et al. (2015) [4] |
| Monitoring warfarin patients undergoing dose initiation in the first 3 months (3-monthly) | 161.81 | Gamma, Mean = 161.81, Standard deviation = 161.81 | Guidelines for the Management of Adult Patients requiring Anticoagulation Treatment with Warfarin [5] |
| Monitoring DOAC patients (3-monthly) | 10.00 | Gamma, Mean = 10.00, Standard deviation = 10.00 | Unit costs of health and social care, 2018 [6] & NUTH costing tool, 2018 [7] |
| Monitoring warfarin in the first 3 months following genetic testing if normal sensitivity | 45.00 | N/A | Janzic et al. (2015) [8] |
| Monitoring warfarin in the first 3 months following genetic testing if moderate/major sensitivity | 90.00 | N/A | Janzic et al. (2015) [8] and You et al. (2012) [3] |
| Self-testing warfarin in year 1 (3-monthly) | 117.71 | Gamma, Mean = 117.71, Standard deviation = 117.71 | Sharma et al. (2015) [4] |
| Self-testing warfarin in subsequent years (3-monthly) | 84.87 | Gamma, Mean = 84.87, Standard deviation = 84.87 | Sharma et al. (2015) [4] |
| Acute MI | 6,017.39 | Gamma, Mean = 6,017.39, Standard deviation = 6,017.39 | Dretzke et al. (2015) [9] |
| Fatal MI | 2,587.03 | Gamma, Mean = 2,587.03, Standard deviation = 2,587.03 | Dretzke et al. (2015) [9] |
| Extracranial haemorrhage | 2,707.70 | Gamma, Mean = 2,707.70, Standard deviation = 2,707.70 | Sharma et al. (2015) [4] |
| Intracranial haemorrhage | 7,953.39 | Gamma, Mean = 7,953.39, Standard deviation = 7,953.39 | Sharma et al. (2015) [4] |
| Minor stroke | 2,316.73 | Gamma, Mean = 2,316.73, Standard deviation = 2,316.73 | NHS Reference costs, 2017/18 [10] |
| Major stroke | 11,663.80 | Gamma, Mean = 11,663.80, Standard deviation = 11,663.80 | Sharma et al. (2015) [4] |
| Fatal stroke | 10,227.48 | Gamma, Mean = 10,227.48, Standard deviation = 10,227.48 | Dretzke et al. (2015) [9] |
| Systemic embolism | 1,811.59 | Gamma, Mean = 1,811.59, Standard deviation = 1,811.59 | Sharma et al. (2015) [4] |
| Post Intracranial haemorrhage (3-monthly) | 2,825.00 | Gamma, Mean = 2,825.00, Standard deviation = 2,825.00 | Sharma et al. (2015) [4] |
| Post minor stroke (3-monthly) | 339.54 | Gamma, Mean = 339.54, Standard deviation = 339.54 | Sharma et al. (2015) [4] |
| Post major stroke (3-monthly) | 4,377.13 | Gamma, Mean = 4,377.13, Standard deviation = 4,377.13 | Sharma et al. (2015) [4] |
| Post MI (3-monthly) | 602.07 | Gamma, Mean = 602.07, Standard deviation = 602.07 | Dretzke et al. (2015) [9] |

**Clinical parameters**

The percentage of patients who are prescribed warfarin for the treatment of atrial fibrillation in current practice was based on UK anticoagulation prescribing data [11]. You et al. [3] provided data on the probabilities of being in range and out of range on warfarin, and the probabilities of being above range and below range when out of range. 3-monthly probabilities of stroke when in range, below range and above range on warfarin and the 3-monthly probability of MI, and death from MI, were derived from You et al. [3]. The 3-monthly probabilities of systemic embolism on warfarin, death following systemic embolism, major bleeding when in range, below range and above range were all derived from previous cost-effectiveness analyses [3, 4, 8]. Probabilities for likelihood that major bleeding is intracranial haemorrhage on warfarin, death following intracranial and extracranial haemorrhage, severity of stroke, likelihood that major bleeding is intracranial haemorrhage on DOACs, probability of MI on DOACs, and probabilities of sensitivity status (to warfarin) following a genetic test, were all derived from You et al. [3]. Subsequent prescribing practice following a genetic test, i.e. percentage of patients prescribed warfarin given sensitivity results, was unknown; therefore, these values were assumed and were varied in sensitivity analysis. Probabilities of being in range on warfarin at different sensitivity levels were derived from You et al. [3]. Where percentage of time in range, and out of range (and when out of range, percentage of time above range and below range) was known, weighted probabilities of clinical events could be estimated using information on the probability of events at each level. As the likelihood of experiencing an adverse clinical event is typically lower when in range compared to likelihood when out of range, increasing percentage of time in range will result in a lower probability of experiencing an adverse event. All clinical probabilities included in the model are shown in Table 2 below.

***Table 2*** ***Base-case probabilities used in the model***

| **Variable** | **Value** | **Distribution** | **Source** |
| --- | --- | --- | --- |
| Probability of being prescribed warfarin with current practice | 0.40 | Beta, α = 2, β = 2 | [www.openprescribing.net](http://www.openprescribing.net) [11] |
| Probability of being in range on warfarin | 0.64 | Beta, α = 3.555556, β = 2 | You et al. (2012) [3] |
| Probability of being out of range on warfarin | 0.36 | N/A | You et al. (2012) [3] |
| Probability of being above range when out of range | 0.48 | Beta, α = 2, β = 2.166667 | You et al. (2012) [3] |
| Probability of being below range when out of range | 0.52 | Beta, α = 2.166667, β = 2 | You et al. (2012) [3] |
| Probability of stroke when INR in range (3-monthly) | 0.003 | Beta, α = 2, β = 610.3764 | You et al. (2012) [3] |
| Probability of stroke when INR below range (3-monthly) | 0.006 | N/A | You et al. (2012) [3] |
| Probability of stroke when INR above range (3-monthly) | 0.003 | N/A | You et al. (2012) [3] |
| Probability of MI on warfarin (3-monthly) | 0.002 | Beta, α = 2, β = 1244.996 | You et al. (2012) [3] |
| Probability of death following MI | 0.15 | Beta, α = 2, β = 11.33333 | You et al. (2012) [3] |
| Probability of systemic embolism on warfarin (3-monthly) | 0.0004 | Beta, α = 2, β = 57009.283 | Janzic et al. (2015) [8] |
| Probability of death following systemic embolism | 0.004 | Beta, α = 2, β = 498 | Sharma et al. (2015) [4] |
| Probability of major bleeding when INR in range on warfarin (3-monthly) | 0.004 | Beta, α = 2, β = 528.3239 | You et al. (2012) [3] |
| Probability of major bleeding when INR below range on warfarin (3-monthly) | 0.004 | N/A | You et al. (2012) [3] |
| Probability of major bleeding when INR above range on warfarin (3-monthly) | 0.03 | N/A | You et al. (2012) [3] |
| Probability that major bleed is intracranial haemorrhage on warfarin | 0.22 | Beta, α = 2, β = 7.090909 | You et al. (2012) [3] |
| Probability of death following intracranial haemorrhage | 0.49 | Beta, α = 2, β = 2.115226 | You et al. (2012) [3] |
| Probability of death following extracranial haemorrhage | 0.05 | Beta, α = 2, β = 37.21569 | You et al. (2012) [3] |
| Probability no deficit after stroke | 0.09 | N/A | You et al. (2012) [3] |
| Probability mild deficit after stroke | 0.425 | N/A | You et al. (2012) [3] |
| Probability major deficit after stroke | 0.402 | N/A | You et al. (2012) [3] |
| Probability that major bleed is intracranial haemorrhage on DOAC | 0.089 | Beta, α = 2, β = 20.47191 | You et al. (2012) [3] |
| Probability of MI on DOAC (3-monthly) | 0.002 | Beta, α = 2, β = 970.6046 | You et al. (2012) [3] |
| Probability major sensitivity following genetic test | 0.066 | Beta, α = 2, β = 28.30303 | You et al. (2012) [3] |
| Probability moderate sensitivity following genetic test | 0.266 | Beta, α = 2, β = 5.518797 | You et al. (2012) [3] |
| Probability normal sensitivity following genetic test | 0.668 | N/A | You et al. (2012) [3] |
| Probability warfarin if normal sensitivity | 0.90 | Beta, α = 18, β = 2 | Assumption |
| Probability warfarin if moderate sensitivity | 0.50 | Beta, α = 2, β = 2 | Assumption |
| Probability warfarin if major sensitivity | 0.10 | Beta, α = 2, β = 18 | Assumption |
| Probability of being in range on warfarin if normal sensitivity | 0.789 | Beta, α = 7.478673, β = 2 | You et al. (2012) [3] |
| Probability of being in range on warfarin if moderate sensitivity | 0.571 | Beta, α = 2.662005, β = 2 | You et al. (2012) [3] / Assumption |
| Probability of being in range on warfarin if major sensitivity | 0.50 | Beta, α = 2, β = 2 | You et al. (2012) [3] / Assumption |

**Relative risks**

In order to adjust the probabilities of adverse events occurring when above or below range, when taking DOACs, or when undergoing self-testing, relative risk values were applied. All relative risks were derived from previous literature [3, 4, 8] and are shown in Table 3 below.

***Table 3*** ***Relative risks***

| **Variable** | **Value** | **Source** |
| --- | --- | --- |
| RR of stroke when on dabigatran 110mg | 1.11 | You et al. (2012) [3] |
| RR of major bleed when on dabigatran 110mg | 0.80 | You et al. (2012) [3] |
| RR of systemic embolism when on dabigatran 110mg | 0.61 | Janzic et al. (2015) [8] |
| RR of death post minor stroke | 2.33 | Sharma et al. (2015) [4] |
| RR of death post major stroke | 4.11 | Sharma et al. (2015) [4] |
| RR of bleed when above range on warfarin | 8.28 | You et al. (2012) [3] |
| RR of bleed when below range on warfarin | 1 | You et al. (2012) [3] |
| RR of stroke when above range on warfarin | 1 | You et al. (2012) [3] |
| RR of stroke when below range on warfarin | 1.7 | You et al. (2012) [3] |
| RR of bleed when self-testing | 0.99 | Sharma et al. (2015) [4] |
| RR of thromboembolic event when self-testing | 0.99 | Sharma et al. (2015) [4] |

**Utilities**

Baseline utility values for atrial fibrillation patients and decrements associated with adverse events were sourced from two UK-based cost-effectiveness analyses in this clinical area [4, 8] and are shown in Table 4 below. Utility values were adjusted to fit 3-monthly time cycles in the model.

***Table 4 Utility values used in the model***

| **Variable** | **Value** | **Distribution** | **Source** |
| --- | --- | --- | --- |
| Atrial fibrillation (age 65-74) | 0.738 | Beta, α = 5.633588, β = 2 | Sharma et al. (2015) [4] |
| Atrial fibrillation (age 75+) | 0.688 | Beta, α = 4.410256, β = 2 | Sharma et al. (2015) [4] |
| Minor stroke (applied as a decrement) | 0.641 | Beta, α = 3.571031, β = 2 | Sharma et al. (2015) [4] |
| Major stroke (applied as a decrement) | 0.189 | Beta, α = 2, β = 5.582011 | Sharma et al. (2015) [4] |
| Decrement MI | 0.12 | Beta, α = 2, β = 14.66667 | Dretzke et al. (2015) [8] |
| Decrement major bleed | 0.18 | Beta, α = 2, β = 9.025358 | Sharma et al. (2015) [4] |
| Decrement systemic embolism | 0.12 | Beta, α = 2, β = 14.80672 | Sharma et al. (2015) [4] |
| Post minor stroke (age 65-74) | 0.71 | Beta, α = 4.896552, β = 2 | Sharma et al. (2015) [4] |
| Post minor stroke (age 75+) | 0.662 | Beta, α = 3.91716, β = 2 | Sharma et al. (2015) [4] |
| Post major stroke | 0.31 | Beta, α = 2, β = 4.451613 | Sharma et al. (2015) [4] |
| Post major bleed | 0.461 | Beta, α = 2, β = 2.338395 | Sharma et al. (2015) [4] |

**References**

1. British National Formulary. BNF Online. (2018). Available from: <http://www.bnf.org/>.
2. Pirmohamed, M., Burnside, G., Eriksson, N., et al. A Randomized Trial of Genotype-­‐Guided Dosing of Warfarin. (2013) New England Journal of Medicine 369(24): 2294-­‐303.
3. You, J.H.S., Tsui, K.K.N., Wong, R.S.M., Cheng, G. (2012) Cost-effectiveness of Dabigatran versus Genotype-Guided Management of Warfarin Therapy for Stroke Prevention in Patients with Atrial Fibrillation. Plos One 7(6): e39640.
4. Sharma, P., Scotland, G., Cruickshank, M., et al. (2015) The clinical effectiveness and cost-effectiveness of point-of-care tests (CoaguCheck system, INRatio2 PT/INR monitor and ProTime Microcoagulation system) for the self-monitoring of the coagulation status of people receiving long-term vitamin K antagonist therapy, compared with standard UK practice: systematic review and economic evaluation. Health Technology Assessment 19(48): 1366-5278.
5. The Newcastle upon Tyne Hospitals NHS Foundation Trust 2016, Guidelines for the Management of Adult Patients requiring Anticoagulation Treatment with Warfarin.
6. Curtis L, Burns A. Unit Costs of Health & Social Care 2018: The University of Kent; 2018.
7. NHS NUTH costing tool, 2018.
8. Janzic, A., Jos, M. (2015) Cost-effectiveness of novel oral anticoagulants for stroke prevention in atrial fibrillation depending on the quality of warfarin anticoagulation control. PharmacoEconomics 33:395-408.
9. Dretzke, J., Riley, R.D., Lordkipanidze, M., et al. (2015) The prognostic utility of platelet function for the detection of ‘aspirin resistance’ in patients with established cardiovascular or cerebrovascular disease: a systematic review and economic evaluation. Health Technology Assessment 19(37): 1366-5278.
10. Department of Health. NHS Reference Costs: Financial Year 2017 to 2018. London: Department of Health; 2018.
11. www.openprescribing.net [Accessed on 25/07/2019).
