# Supplementary material for: Optimising clinical effectiveness and quality along the atrial fibrillation anticoagulation pathway: an economic analysis
Source: BMC Health Serv Res. 2019 Dec 28;19:1007. doi: 10.1186/s12913-019-4841-3 (PMC6935474; doi:10.1186/s12913-019-4841-3)

Supplementary Information 4

**Figure 1 Economic model to estimate the cost-effectiveness of a redesigned treatment pathway for patients with Atrial Fibrillation**


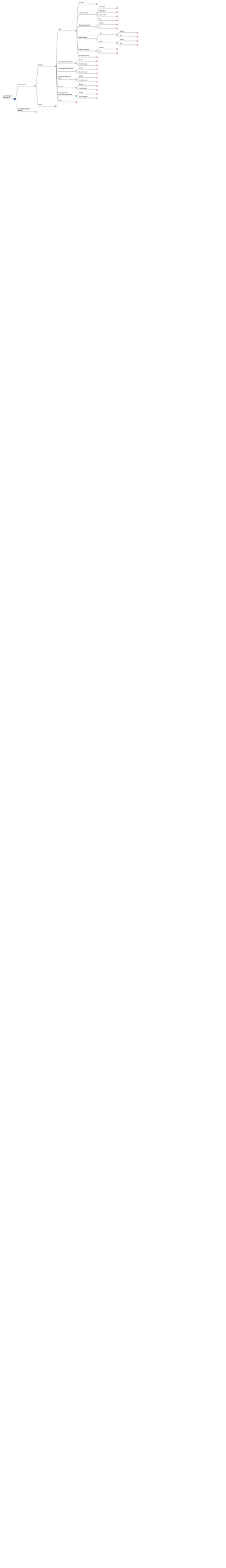

Supplement: Supplementary file 4 — Additional file 4: Figure S1. Economic model to estimate the cost-effectiveness of a redesigned treatment pathway for patients with Atrial Fibrillation. [file 12913_2019_4841_MOESM4_ESM.docx]
