# Supplementary material for: Optimising clinical effectiveness and quality along the atrial fibrillation anticoagulation pathway: an economic analysis
Source: BMC Health Serv Res. 2019 Dec 28;19:1007. doi: 10.1186/s12913-019-4841-3 (PMC6935474; doi:10.1186/s12913-019-4841-3)
Supplement: Supplementary file 5 — Additional file 5: Figure S2. Cost-effectiveness acceptability curve of redesigned treatment pathway vs. current practice: base-case analysis. [file 12913_2019_4841_MOESM5_ESM.docx]

Supplementary Information 5

**Figure 2 Cost-effectiveness acceptability curve of redesigned treatment pathway vs. current practice: base-case analysis**

**
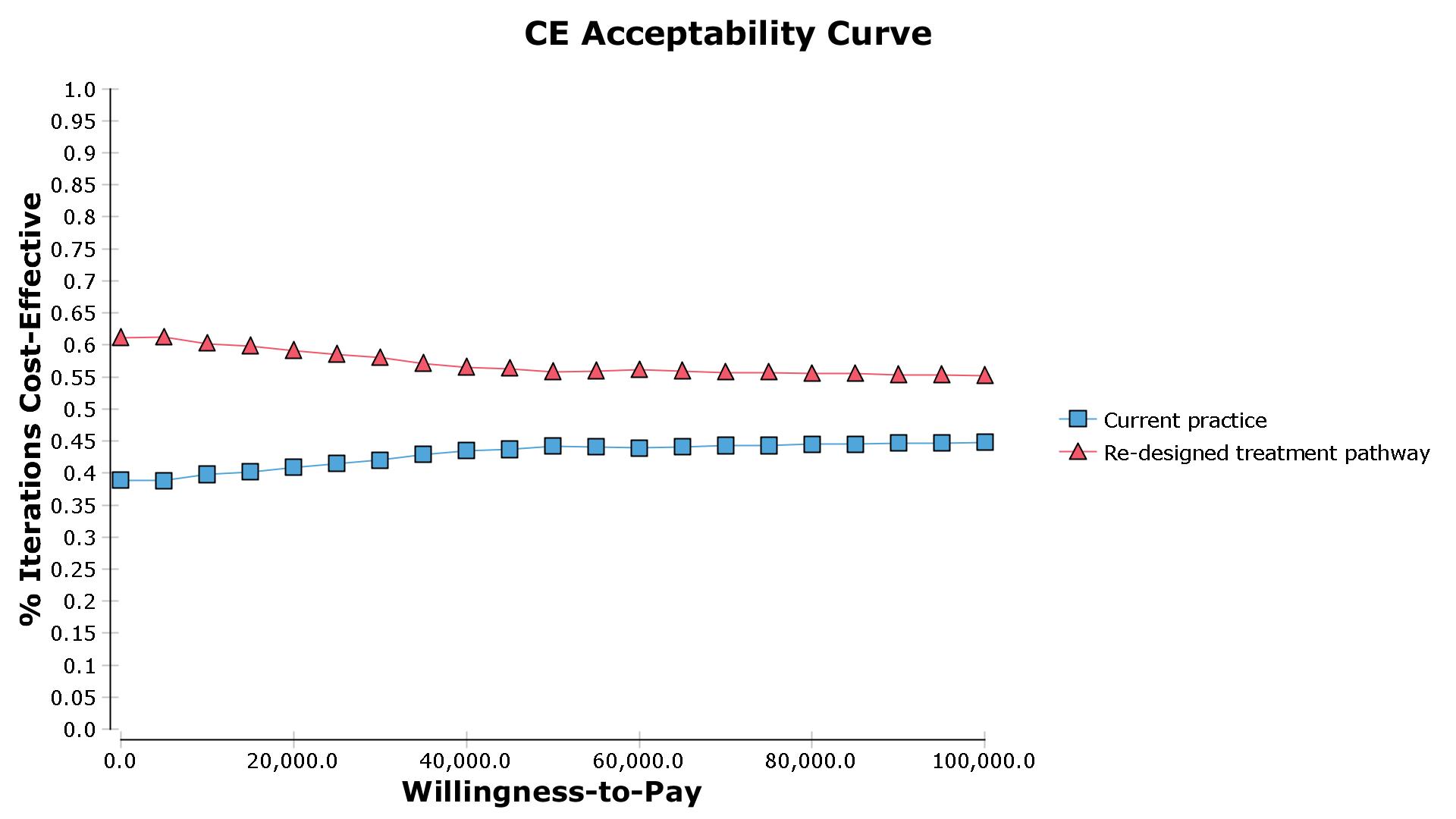
**
