# Supplementary material for: Optimising clinical effectiveness and quality along the atrial fibrillation anticoagulation pathway: an economic analysis
Source: BMC Health Serv Res. 2019 Dec 28;19:1007. doi: 10.1186/s12913-019-4841-3 (PMC6935474; doi:10.1186/s12913-019-4841-3)
Supplement: Supplementary file 6 — Additional file 6: Figure S3. Cost-effectiveness plane of redesigned treatment pathway vs. current practice: base-case analysis. [file 12913_2019_4841_MOESM6_ESM.docx]

Supplementary Information 6

**Figure 3** **Cost-effectiveness plane of redesigned treatment pathway vs. current practice: base-case analysis**

**
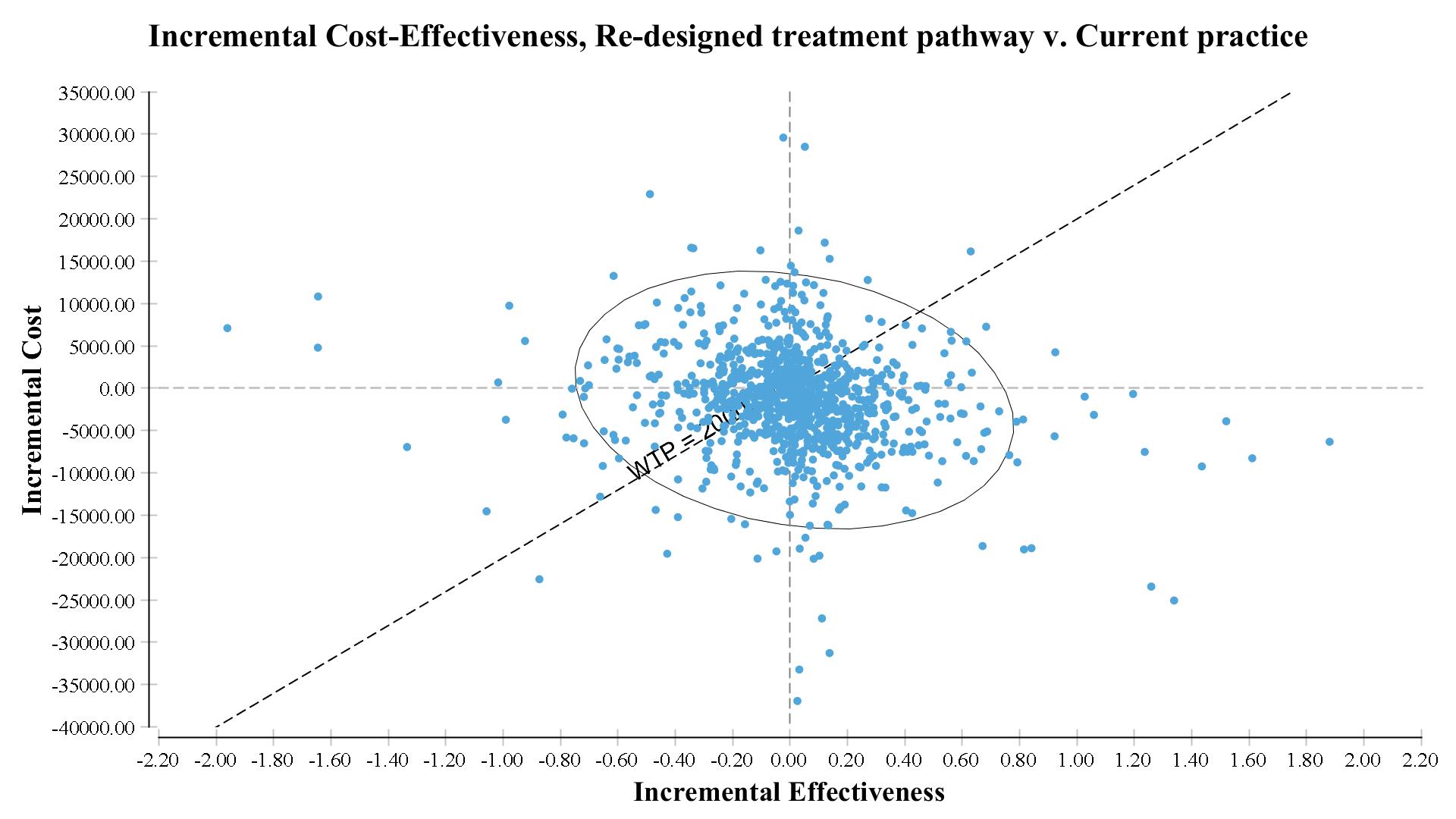
**
